# Supplementary material for: Exploration of the Cs Trapping Phenomenon by Combining Graphene Oxide with α-K6P2W18O62 as Nanocomposite
Source: Materials (Basel). 2021 Sep 26;14(19):5577. doi: 10.3390/ma14195577 (PMC8509777; doi:10.3390/ma14195577)
Supplement: Supplementary file 1 [file materials-14-05577-s001.zip › materials-1367355-supplementary.pdf]

materials

Supplementary materials

Exploration of the Cs Trapping Phenomenon by Combining Graphene Oxide with α-$K_6P_2W_{18}O_{62}$ as Nanocomposite

Bangun Satrio Nugroho [1,2], Akane Kato [2], Chie Kowa [2], Tomoya Nakashima [2], Atsushi Wada [2], Muh. Nur Khoiru Wihadi [3,4] and Satoru Nakashima [1,2,5,*]

[1] Radioactivity Environmental Protection Course, Phoenix Leader Education Program, Hiroshima University, 1-1-1 Kagamiyama, Higashi-Hiroshima 739-8524, Japan; d192886@hiroshima-u.ac.jp

[2] Department of Chemistry, Graduate School of Science, Hiroshima University, 1-3-1 Kagamiyama, Higashi-Hiroshima 739-8526, Japan; kato.akane@jaea.go.jp (A.K.); c.k_0715_titty@icloud.com (C.K.); hakatomo0601@gmail.com (T.N.); m204479@hiroshima-u.ac.jp (A.W.)

[3] Department of Chemistry, Faculty of Military Mathematics and Natural Science, The Republic of Indonesia Defense University, Bogor 16810, Indonesia; nur.wihadi@idu.ac.id

[4] National Research and Innovation Agency (Badan Riset dan Inovasi Nasional), The Republic of Indonesia, Kawasan Puspiptek, Serpong, Tangerang 15314, Indonesia

[5] Natural Science Center for Basic Research and Development, Hiroshima University, 1-4-2 Kagamiyama, Higashi-Hiroshima 739-8526, Japan

* Correspondence: snaka@hiroshima-u.ac.jp

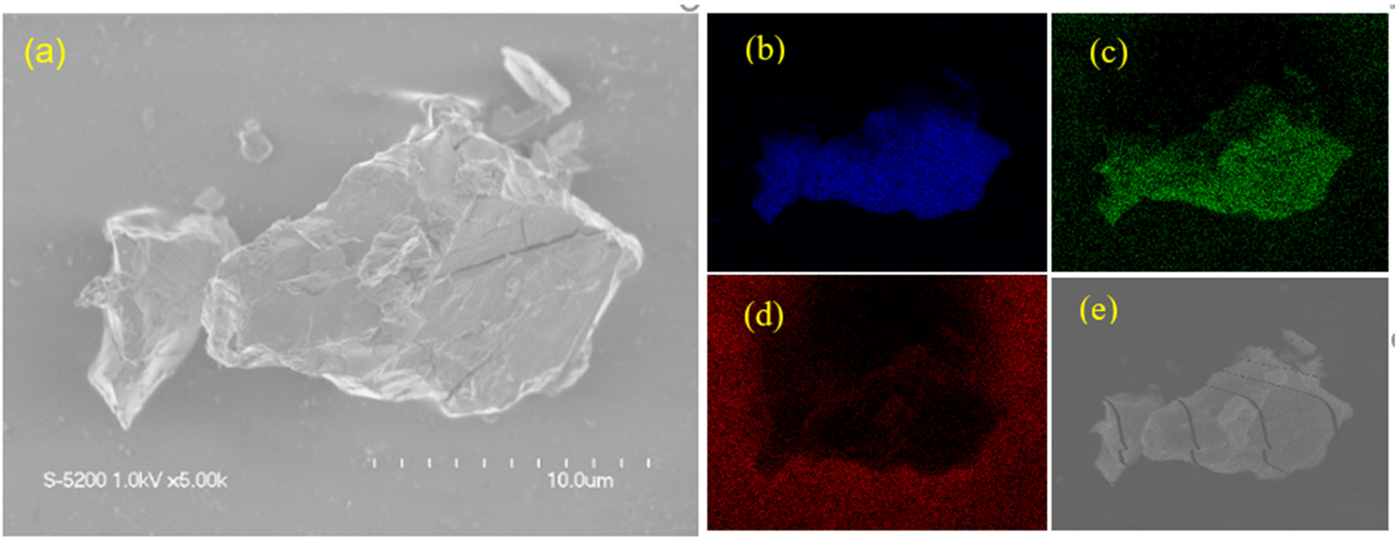

Figure S1. SEM measurement (**a**) α-$K_6P_2W_{18}O_{62}.14H_2O$ (**b**) Tungsten element (**c**) Oxygen element (**d**) Carbon K- α X-ray element (**e**) Secondary electron image.

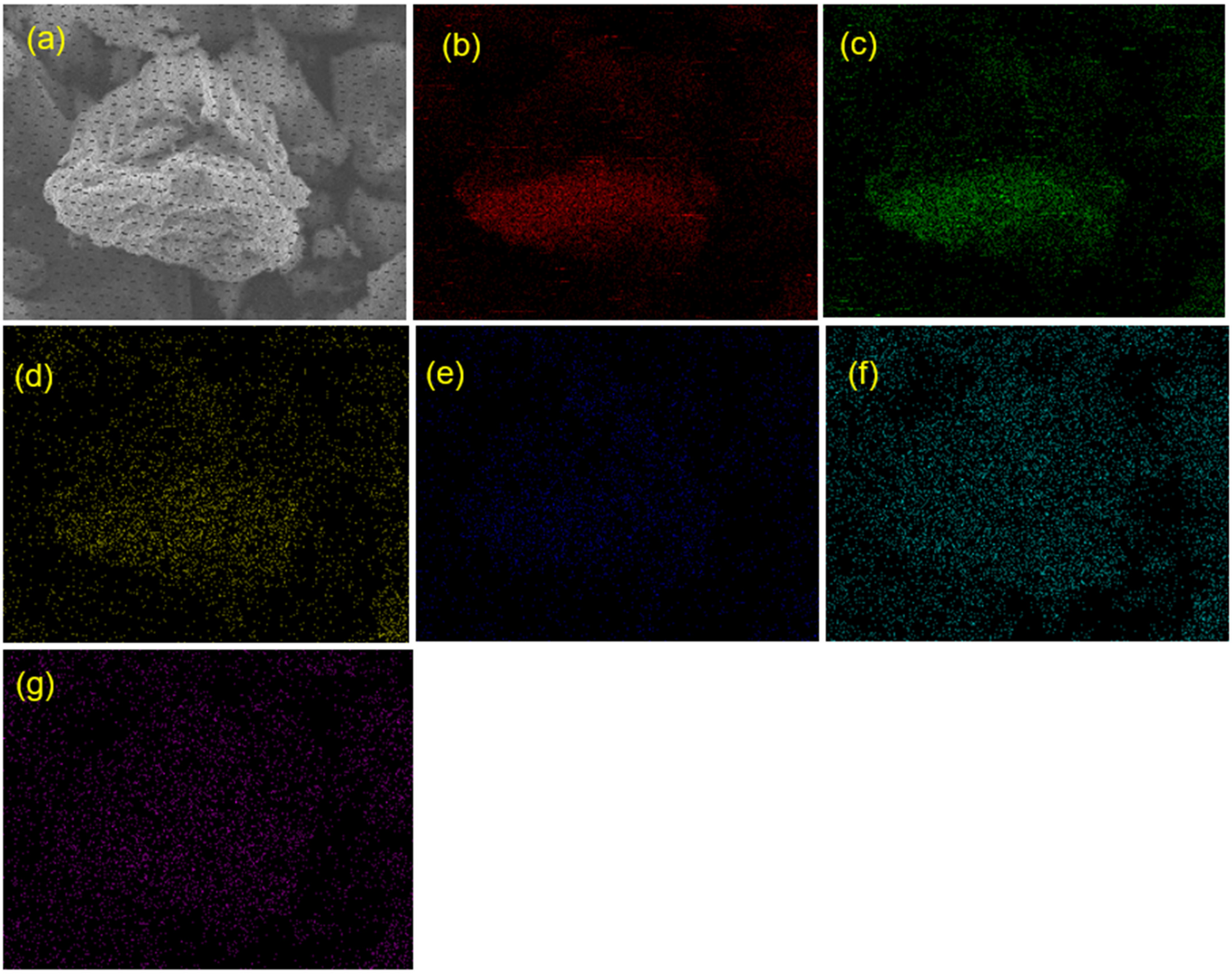

Figure S2. SEM measurement, $[GO_{70}POM]_{41}$ (**a**) Secondary electron image (**b**) Carbon K- α X-ray element (**c**) Oxygen element (**d**) Tungsten element (**e**) Aluminum element (**f**) Cesium element (**g**) Chloride element.

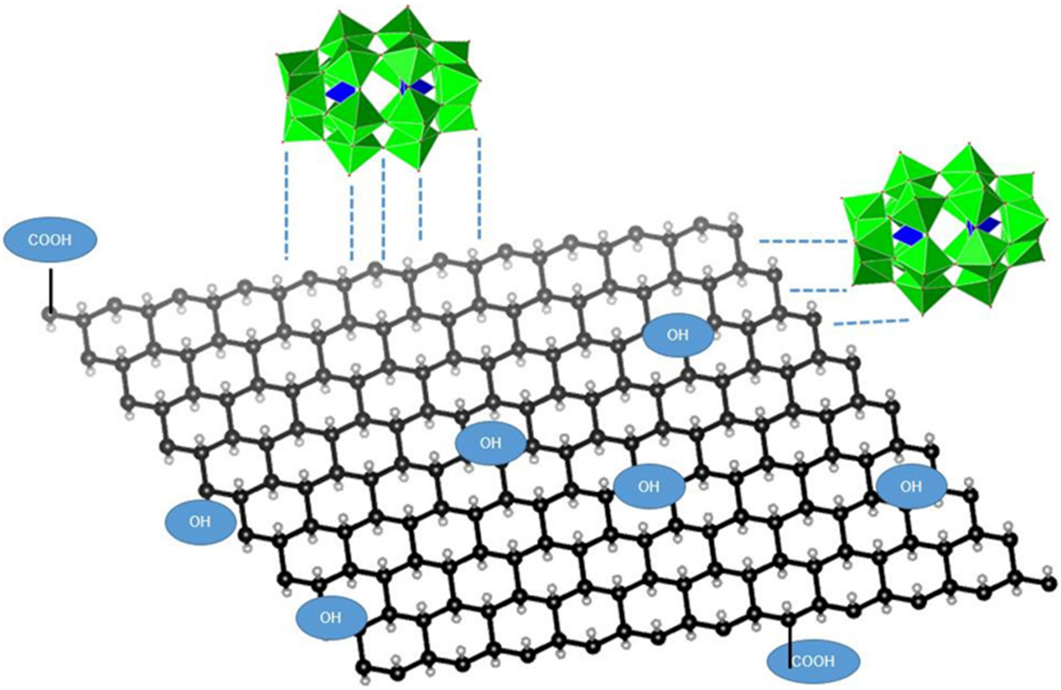

Figure S3. Plausible Interaction between Graphene-Oxide (GO) and Dawson-type Polyoxometalates (POMs). The black and the grey balls corresponds to carbon and hydrogen. The polyhedral model represents to Dawson-type polyoxometalates. The blue dashes indicated of the presence of interaction between GO-POMs.

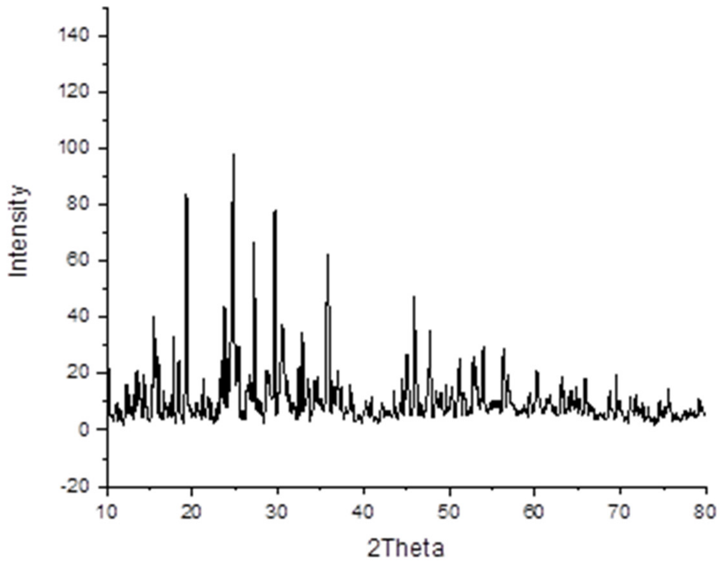

Figure S4. Xrd pattern of α-$K_6P_2W_{18}O_{62}.14H_2O$.

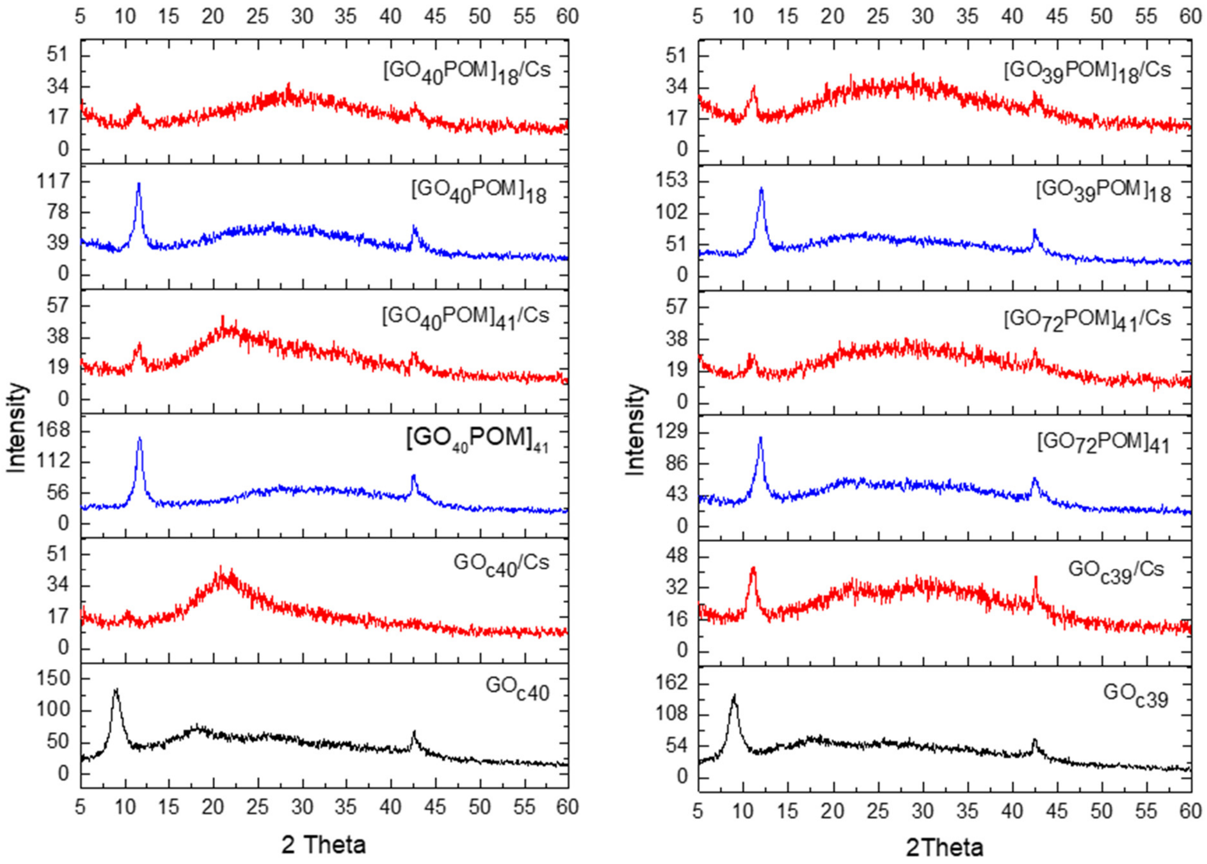

Figure S5. XRD pattern of GO_{c39}, GO_{c40}, GOPOM composite before and after Cs adsorption.

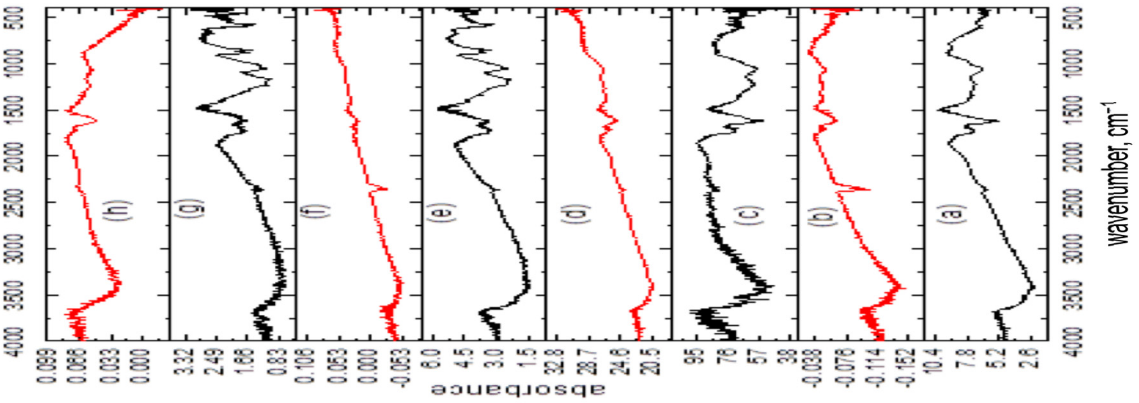

Figure S6. IR spectra for (**a**) GO_{c70} (**b**) GO_{c72} after Cs adsorption (**c**) GO_{c72} (**d**) GO_{c72} after Cs adsorption (**e**) GO_{c39} (**f**) GO_{c39} after Cs adsorption (**g**) GO_{c40} (**h**) GO_{c40} after Cs adsorption.

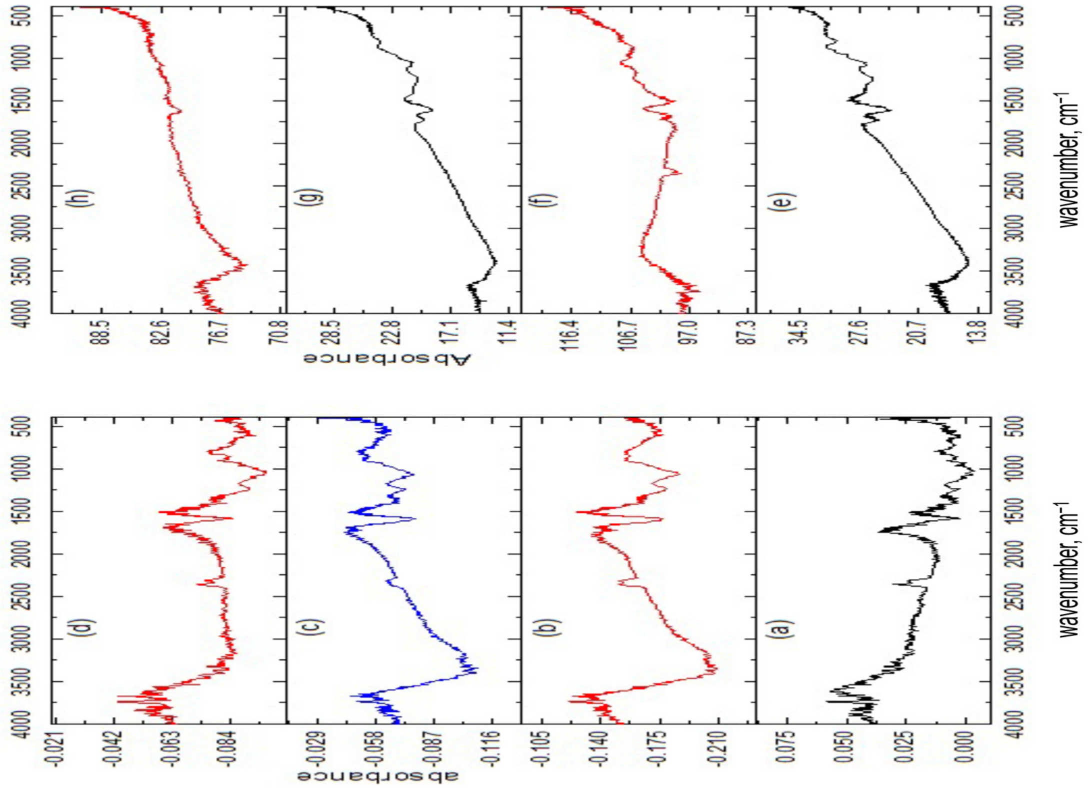

Figure S7. IR spectra for (a) $[GO_{39}POM]_{18}$ (b) $[GO_{39}POM]_{18}$ after Cs adsorption (c) $[GO_{39}POM]_{41}$ (d) $[GO_{39}POM]_{41}$ after Cs adsorption (e) $[GO_{40}POM]_{18}$ (f) $[GO_{40}POM]_{18}$ after Cs adsorption (g) $[GO_{40}POM]_{41}$ (h) $[GO_{40}POM]_{41}$ after Cs adsorption.

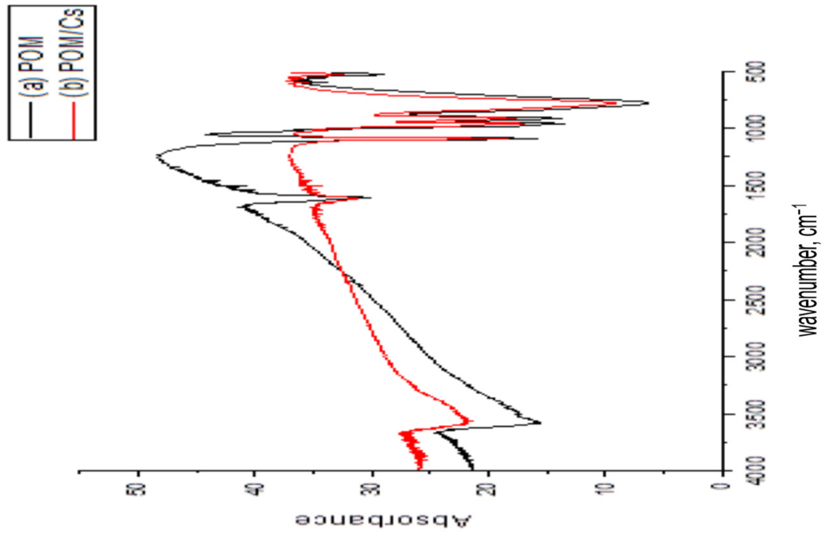

Figure S8. IR spectra for (a)POM (b)POM after Cs adsorption.

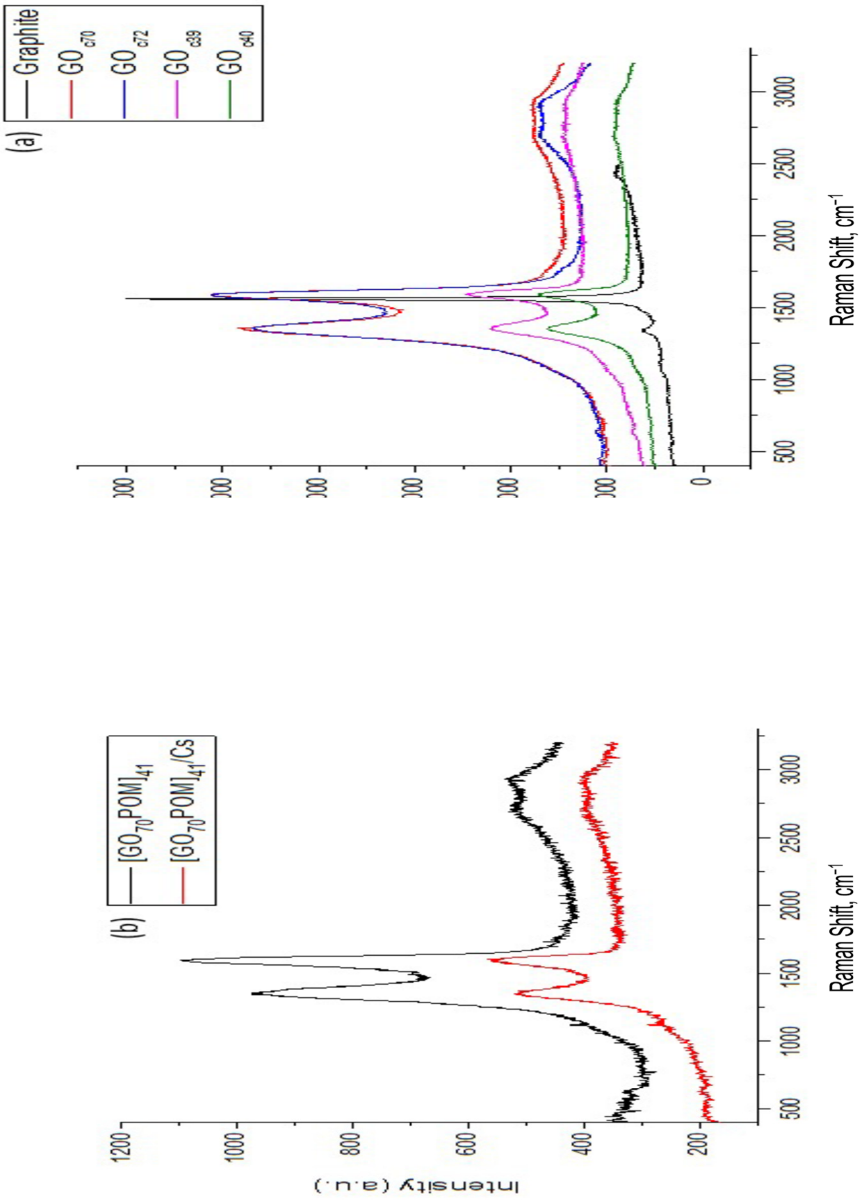

Figure S9. Raman spectra (**a**) Graphite, GO_{c70}, GO_{c72}, GO_{c39}, GO_{c40}, (**b**) $[GO_{70}POM]_{41}$ and after Cs adsorption.

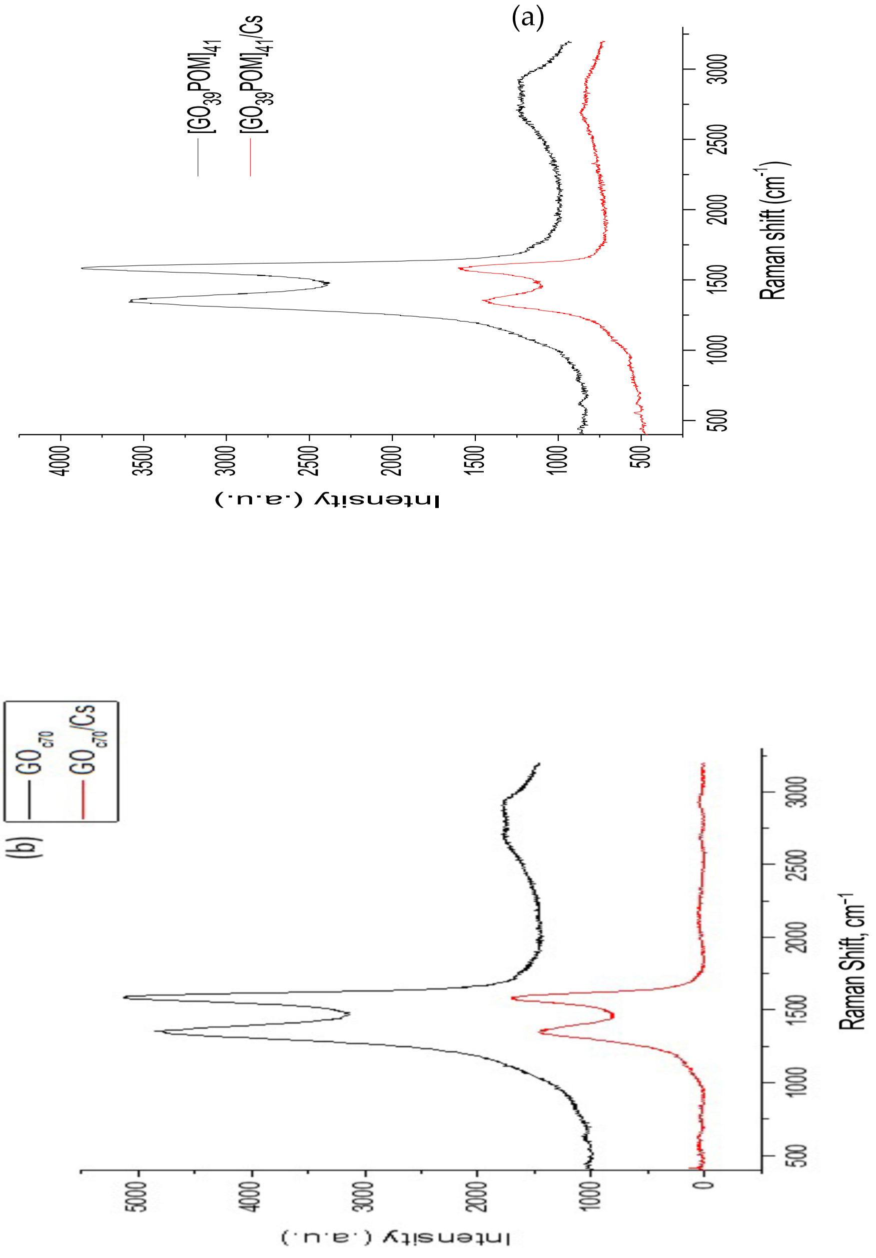

Figure S10. Raman spectra composite (**a**) $[GO_{39}POM]_{41}$ before and after Cs adsorption (**b**) GO_{c70} before and after Cs adsorption.

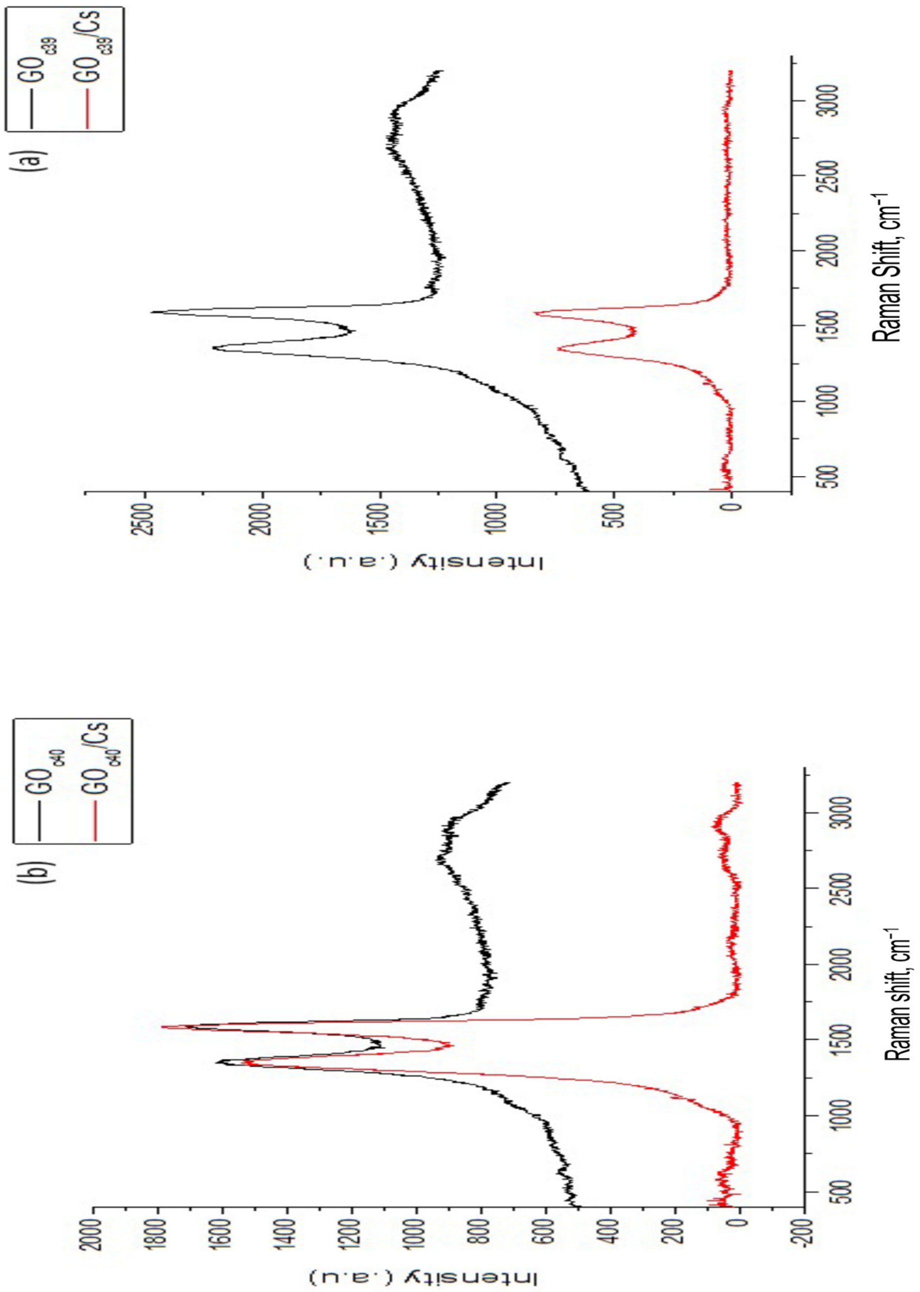

Figure S11. Raman spectra (**a**) GO_{c39} before and after Cs adsorption (**b**) GO_{c40} before and after Cs adsorption.

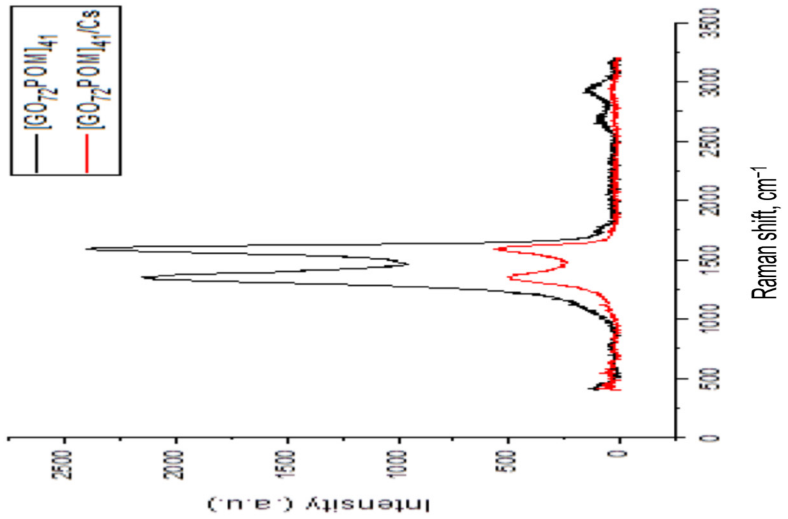

Figure S12. Raman spectra composite $[GO_{72}POM]_{41}$ before and after Cs adsorption.

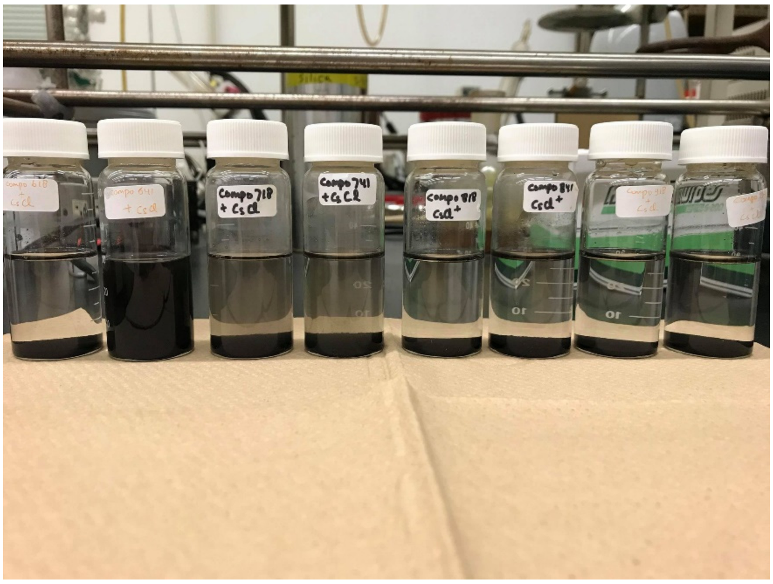

Figure S13. The composite solution after Cs adsorption. Only Composite $[GO_{70}POM]_{41}$ (**b**) that forms stable solution (black color). Another composite solution shows remains coagulated. The photo was taken after more than one-hour stay at ambient temperature.

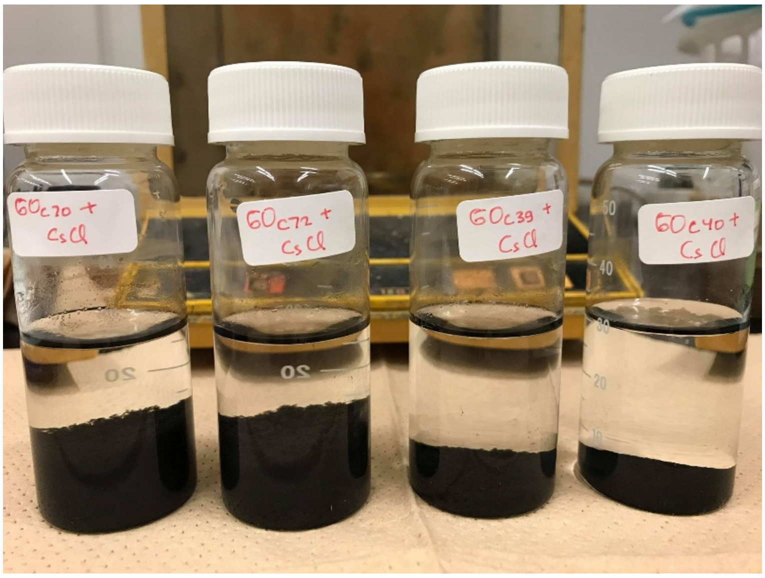

Figure S14. The GO solution after Cs adsorption. All GO solutions shows remains coagulated. (**a**) GO_{c70} and (**b**) GO_{c72} shows different coagulated behavior with (**c**) GO_{c39} and (**d**) GO_{c40}. The photo was taken after more than one-hour stay at ambient temperature.

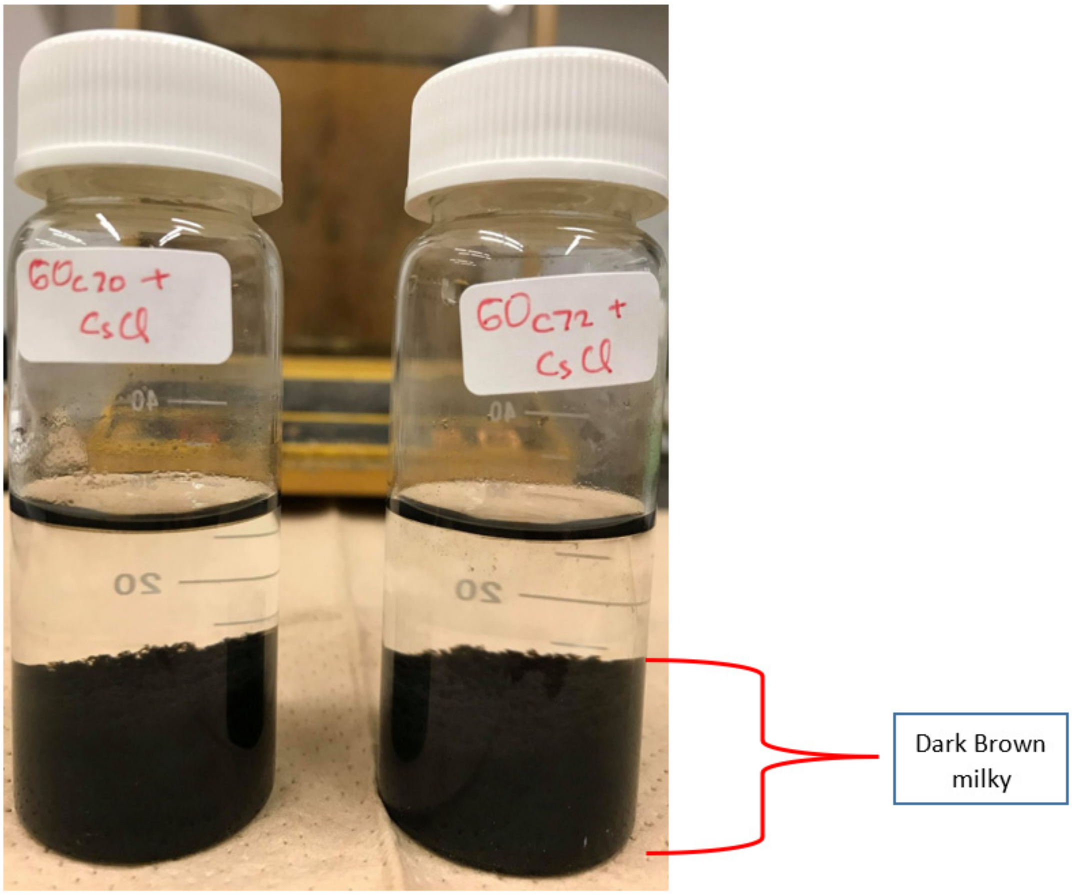

Figure S15. Coagulation process after Cs interaction with (a) GO_{c70} and (b) GO_{c72}. The photo was taken after more than one-hour stay at ambient temperature.

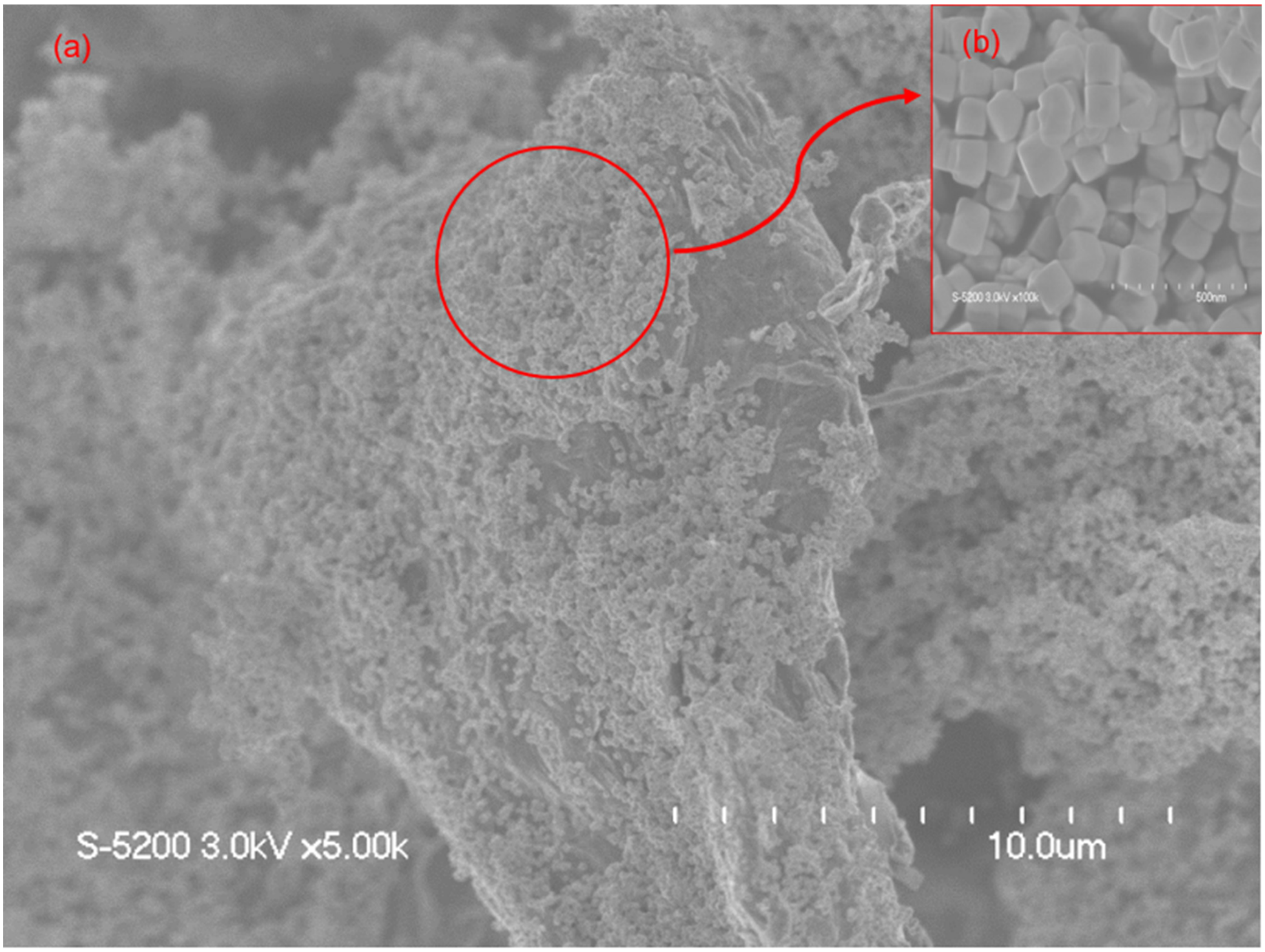

Figure S16. SEM image by using composite (a) $[GO_{70}POM]_{41}$ after Cs adsorption and (b) Cs particle (zoom mode based on figure a).

Table S1. The ratio I_D/I_G of Graphene oxide and composite before and after Cs adsorption.

No.	Material	D band	G band	Ratio I_D/I_G
1	GO_{c70}	1356	1586	0.93
2	GO_{c72}	1352	1581	0.91
3	GO_{c39}	1352	1590	0.89
4	GO_{c40}	1347	1590	0.88
5	$[GO_{39}POM]_{41}$	1347	1581	0.92
6	$[GO_{39}POM]_{41}/Cs$	1352	1586	0.90
7	$[GO_{70}POM]_{18}$	1347	1586	0.96
8	$[GO_{70}POM]_{18}/Cs$	1352	1577	0.92
9	$[GO_{70}POM]_{41}$	1357	1592	0.88
10	$[GO_{70}POM]_{41}/Cs$	1343	1602	0.93

Table S2. The crystallize size of GO.

Sample	Concentration ratio GO:POM	2Φ (°)	FWHM (ß)	Out of plane crystallite size – D (nm)	d (nm)	D (nm) after Cs adsorption	Layer (n)	In plane crystallite size – L (nm)
Graphite	-	26.6	0.28	30.49	0.34	-	90.67	10.27
GO_{c70}	-	11.58	1.93	4.3	0.77	-	6.58	2.59
GO_{c72}	-	8.66	1.69	4.9	1.02	-	5.80	2.53
GO_{c39}	-	8.91	1.57	5.3	0.99	-	6.19	2.56
GO_{c40}	-	9.13	1.47	5.7	0.97	-	6.75	2.61
$[GO_{70}POM]_{18}$	1:8	12.28	1.63	5.12	0.72	0.79	-	2.54
$[GO_{70}POM]_{41}$	4:1	10.99	1.48	5.64	0.81	0.86	-	2.61
$[GO_{72}POM]_{18}$	1:8	12.75	1.10	7.60	0.69	0.77	-	3.04
$[GO_{72}POM]_{41}$	4:1	12.05	1.07	7.80	0.73	0.79	-	3.09
$[GO_{39}POM]_{18}$	1:8	11.92	1.19	7.02	0.74	0.81	-	2.89
$[GO_{39}POM]_{41}$	4:1	11.86	1.07	7.80	0.74	0.79	-	3.09
$[GO_{40}POM]_{18}$	1:8	11.57	0.84	9.94	0.76	0.83	-	3.71
$[GO_{40}POM]_{41}$	4:1	11.57	0.24	34.78	0.76	0.78	-	11.95

*FWHM was calculated by using origin software.
